# A novel cytoprotective organ perfusion platform for reconstructing homeostasis of DCD liver while alleviating IRI injury

**DOI:** 10.1002/btm2.10724

**Published:** 2024-09-23

**Authors:** Tingting Lan, Mingxing Yu, Tao Ming, Hong Wang, Juan Deng, Shuhan Cheng, Zhongyang Shen, Deling Kong

**Affiliations:** ^1^ Research Institute of Transplant Medicine, Tianjin First Central Hospital, School of Medicine, Nankai University Tianjin China; ^2^ State Key Laboratory of Medicinal Chemical Biology College of Life Science, Nankai University Tianjin China; ^3^ Institute of Biomedical Engineering, Chinese Academy of Medical Sciences & Peking Union Medical College Tianjin China

**Keywords:** normothermic machine perfusion systems, organ perfusion and transplantation, ventricular assist devices

## Abstract

Pump is a vital component for expelling the perfusate in small animal isolated organ normothermic machine perfusion (NMP) systems whose flexible structure and rhythmic contraction play a crucial role in maintaining perfusion system homeostasis. However, the continuous extrusion forming with the rigid stationary shaft of the peristaltic pumps can damage cells, leading to metabolic disorders and eventual dysfunction of transplanted organs. Here, we developed a novel biomimetic blood‐gas system (BBGs) for preventing cell damage. This system mimics the cardiac cycle and features an adjustable inspiratory‐to‐expiratory (IE) ratio to mitigate acidosis caused by continuous oxygen inhalation. In our study, adipose stem cells (ADSCs) were cultured within the circulatory system for 10 min, 2, and 4 h. Compared to the peristaltic pump, the BBGs significantly reduced cell apoptosis and morphological injury while enhancing cell proliferation and adhesion. Additionally, when the supernatant from ADSCs was introduced to LPS‐induced macrophages for 24 h, the BBGs group demonstrated a more pronounced anti‐inflammatory effect, characterized by reduced M1 macrophage expression. Besides, with isolated rat livers from donation after circulatory death (DCD) perfusion with ADSCs for 6 h by the BBGs, we detected fewer apoptotic cells and a reduced inflammatory response, evidenced by down‐regulated TNF‐α expression. The development of BBGs demonstrates the feasibility of recreating physiological liquid–gas circulation in vitro, offering an alternative platform for isolated organ perfusion, especially for applications involving cell therapy.

**Translational Impact Statement**

The ex vivo organ perfusion system, pivotal for enhancing long‐term organ preservation and salvaging marginal donors, particularly in small animal models, has been significantly advanced through the development of a biomimetic blood‐gas system (BBGs) in this article. This system characterizes by a flexible balloon pump and a simulated cardiac rhythm and a ventilation device that mimics lung function, ensuring prefer cellular integrity. We apply this design for mitigating the injury in donation after circulatory death (DCD) livers by preserving the therapeutic attributes of adipose‐derived stem cells (ADSCs).

## 1 INTRODUCTION

As the representative of the ventricular assist devices (VADs) during extracorporeal circulation, centrifugal pumps have become the greatest important life‐saving components for patients suffering from heart failure,^1^, ^2^, ^3^, ^4^ assuming the role of the heart in facilitating blood circulation. However, even for HeartMate 3, which has been the world widely smallest and most advanced portable centrifugal pump,^5^, ^6^, ^7^ it still fails to satisfy the requirements of low‐flow velocity below 5 mL/min.^8^, ^9^ Consequently, peristaltic pumps, which feature mechanically rigid components and operate by squeezing perfusate through a soft tube and stationary shaft, have become prevalent in VADs for the extracorporeal perfusion of isolated organs in small animals. These pumps are favored for their cost‐effectiveness, mechanical stability, and precise flow control.^10^, ^11^ Nonetheless, the continuous squeezing action inherent in peristaltic pumps imposes detrimental effects on cellular entities, disrupting the microenvironment within the perfusion system.^12^ The mechanical action of peristaltic pumps can cause the rupture of red blood cells (RBCs), releasing hemoglobin and lactic dehydrogenase (LDH).^13^, ^14^ This damage reduces the oxygen‐carrying capacity of RBCs, leading to organ hypoxia and the accumulation of lactic acid (LA), which in turn causes oxidative stress (OS).^15^, ^16^ Additionally, the release of numerous pro‐inflammatory factors from ruptured cells inflicts tissue damage and exacerbates ischemia–reperfusion injury (IRI).^17^ Many current studies continuously oxygenate perfusion fluid for increasing dissolved oxygen concentration. However, a balance between oxygen demand and consumption as the natural function of RBCs in supplying oxygen can always not be achieved. Actually, continuous oxygen supply in the perfusion system inadvertently result in overventilation, leading to oxygen toxicity and excessive release of reactive oxygen species (ROS) within cells. This process causes acidosis and further exacerbates IRI.^15^, ^16^, ^17^, ^18^, ^19^, ^20^ Hence, reengineering the extracorporeal organ perfusion system to enhance cytocompatibility and biochemical stability is critically important.

Organ transplantation represents the optimal end‐stage treatment for numerous acute and chronic diseases. Nevertheless, the limited availability of donor organs significantly restricts the feasibility of this therapy.^21^ Therefore, an urgent need exists for novel approaches to extend the preservation time of donated organs, especially those from marginal donors such as those following circulatory death, and to restore their function. Donation after Circulatory Death (DCD) involves harvesting organs from individuals who have experienced irreversible cardiac and respiratory arrest. DCD donors often exhibit compromised organ quality due to microcirculatory impairment, cellular apoptosis, and elevated rates of delayed graft function (DGF) or early failure.^22^ These clinically categorize them as extended criteria or marginal donors, necessitating specialized assessment protocols divergent from standard criteria. Over the past decade, DCD liver acceptance rates in eight active programs countries have varied from 18.9% to 74.2%, averaging approximately 28.5%.^23^ Recent advancements in NMP technology have standardized DCD liver quality evaluation, facilitated more precise clinical judgment and reduced DGF incidences.^24^ This innovation enhances the utilization and efficacy of DCD organs, optimizing outcomes in transplantation medicine.

Stem cells (SCs), characterized as primitive or immature cells, can be guided to differentiate into various cell types owing to their self‐renewal and multi‐directional differentiation potential.^25^ This makes them a promising alternative for the repair or reconstruction of damaged or lost tissues and organs. Previous studies have demonstrated that mesenchymal stem cells (MSCs), including both bone marrow‐derived MSCs (BMSCs) and adipose‐derived stem cells (ADSCs), have a positive impact on reducing apoptotic cells and mitigating inflammation levels in DCD livers, thereby significantly alleviate oxidative stress (OS) injuries.^26^, ^27^, ^28^ As a crucial type of stem cell, adipose‐derived stem cells (ADSCs) impact inflammatory cells both directly and indirectly by secreting anti‐inflammatory factors and exosomes containing mRNA, miRNA, and rRNA.^29^, ^30^ In tissue repair, ADSCs release a range of bioactive factors, including transforming growth factor‐β (TGF‐β), hepatocyte growth factor (HGF), insulin‐like growth factor‐1 (IGF‐1), platelet‐derived growth factor (PDGF), and vascular endothelial growth factor (VEGF).^31^, ^32^ Additionally, ADSCs can differentiate into various functional cells at injury sites, such as adipocytes, osteocytes, and chondrocytes, thereby promoting tissue repair and regeneration.^29^ Therefore, it is crucial to preserve the biological properties of perfused SCs, including ADSCs, for the effective preservation and repair of the isolated organs.

In this study, a biomimetic blood‐gas system (BBGs) that closely mimicking physiological metabolism was designed and fabricated. It features a pump‐out design with a flexible one‐way valve controlled by programming with a similarity of the cardiac cycle with that in vivo and an adjustable inspiratory‐to‐expiratory (IE) ratio to alleviate blood gas disorders resulting from continuous oxygen inhalation. By employing mechanical engineering techniques to mimic the systolic and diastolic functions of the heart, we gain precise control over the velocity and frequency of the perfusate from the pump. Furthermore, a proper ventilation/perfusion balance was maintained by adjusting the IE ratio of oxygen through a ventilator. We further evaluated the impact of the newly constructed BBGs on the biological properties of rat‐derived ADSCs and the therapeutic effect of ADSCs on DCD liver. Our findings indicate that the BBGs significantly mitigates the mechanical damage to circulating ADSCs, preserving cytoskeleton integrity, migratory and adhesive capacities, and inhibiting inflammatory responses. Consequently, this leads to a reduction in IRI of DCD livers.

## 2 RESULTS

### 2.1 Development of biomimetic blood‐gas system

**FIGURE 1:**
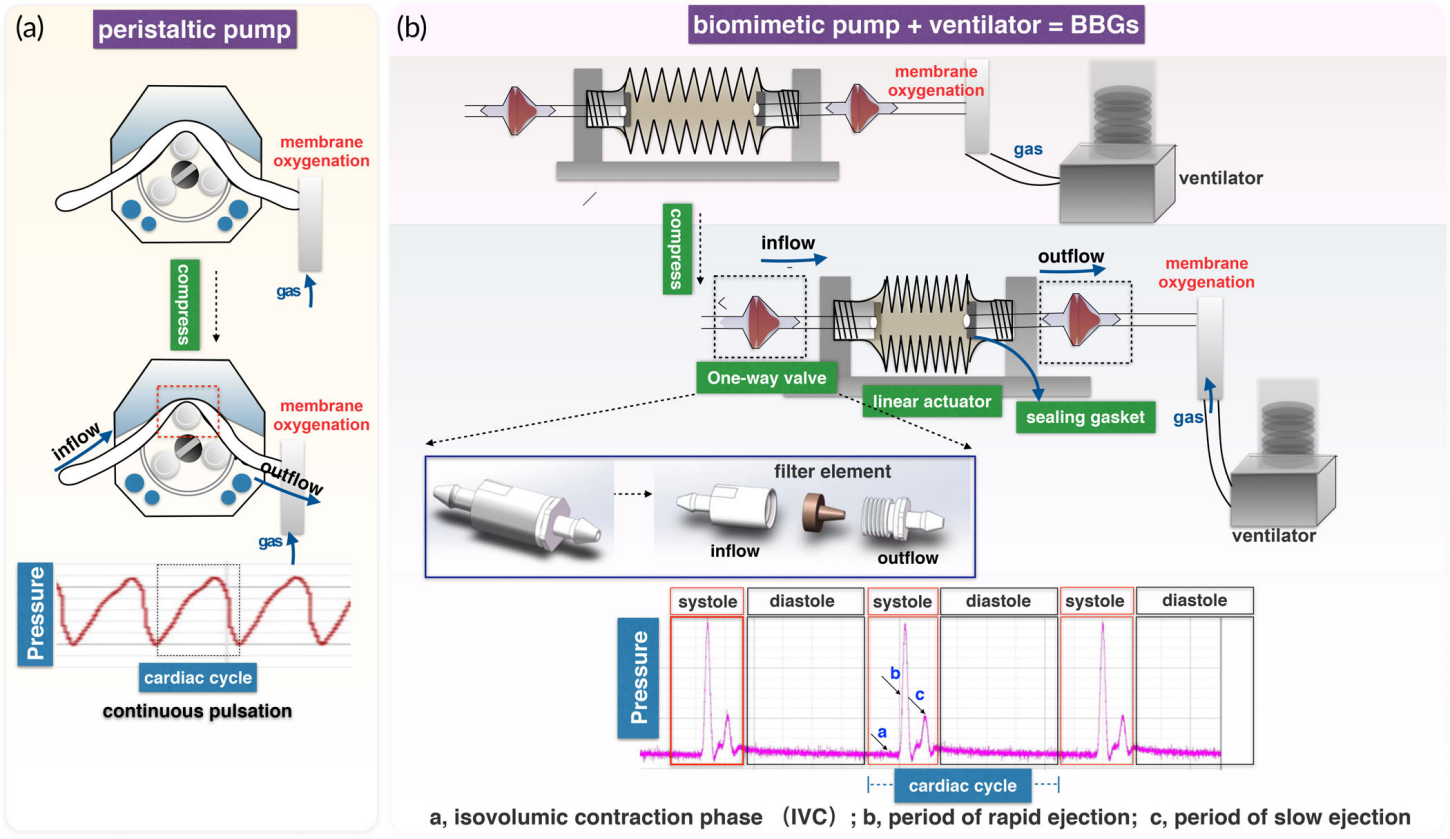
Peristaltic pump and BBGs fabrication. (a) Schematic of the peristaltic pump. (b) Schematic of BBGs.

We developed BBGs based on the bionic principle of heart squeeze pumping and simulated heartbeat and lung respiratory rhythm modules, using 3D printing technology. However, a peristaltic pump can only generate a single sine wave (Figure 1a). We designed a program to simulate different phases of the cardiac cycle in a healthy adult with a heart rate of 60 beats per minute. The program includes a 0.39‐s systolic phase and a 0.66‐s diastolic phase to simulate the systolic and diastolic patterns of the human heart. The systolic stage contains a 0.063‐s isovolumetric contraction (IVC) phase with no outflow, a 0.113‐s rapid ejection period that pumps out 80% of the flow, and a 0.163‐s slow ejection period that pumps out the remaining 20% (Figure 1b). Furthermore, we integrated a ventilator into the BBGs to closely simulate physiological respiratory states by controlling the inhalation/exhalation ratio and the tidal volume of oxygen. This is crucial for maintaining blood gas and acid–base equilibrium within the perfusion system.

### 2.2 Perfusion system based on BBGs provided a more stable environment for isolated organs

**FIGURE 2:**
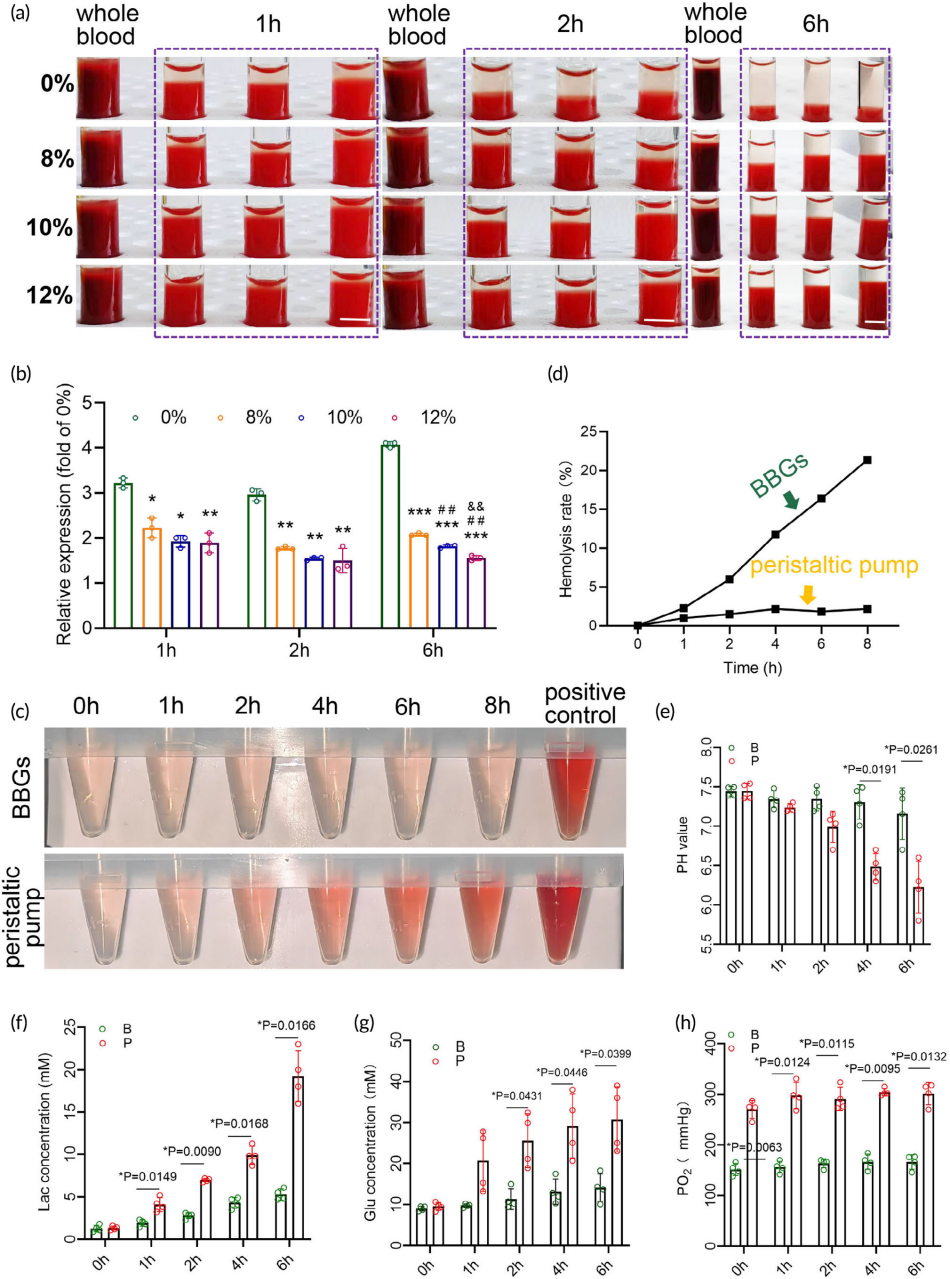
Perfusion system based on BBGs provided a more stable environment for isolated organs. (a) General view of ESR and (b) relative ESR expression of the perfusate with extra 8%, 10%, and 12% dextran‐40 added at 1, 2, and 6 h at room temperature. *n* = 3. (c) Relative hemolysis rate of the perfusate by biomimetic pump or peristaltic pump, and (d) general view of hemolysis at a flow of 5 mL/min at 1, 2, 4, 6, and 8 h. Positive control: 10% whole blood diluted with distilled water; (e) pH value, (f) lactic acid concentration, (g) glucose concentration, and (h) oxygen partial pressure (PaO_2_) of the extracorporeal liver perfusion system at 0, 1, 2, 4, and 6 h by BBGs or peristaltic pump at a flow of 5 mL/min. *n* = 4. B, BBGs; P, peristaltic pump. Scale bars, 6 mm. Data represents the mean ± SD. * versus 0%, *p < 0.05, **p < 0.01, ***p < 0.001; ^#^ versus 8%, ^##^ *p* < 0.01; ^&^ versus 10%, ^&&^ *p* < 0.01.

The infusion formulations used in NMP typically include RBCs for oxygen transport. However, perfusion systems using peristaltic pumps frequently encounter issues such as cell sedimentation, entrapment, and fragmentation caused by the physical compression principle. To evaluate the stability of RBCs under perfusion conditions, we performed erythrocyte sedimentation rate (ESR) and hemolysis tests. In vitro hemodilution increased the ESR due to enhanced aggregation forces among RBCs (Figure 2a). As a result, erythrocytes tended to settle within pipelines or the perfusate container under low flow velocity conditions, typically less than 5 mL/min during isolated organ perfusion in small animals. This prevented erythrocytes from effectively entering the perfusion cycle and performing their oxygenation function. Under physiological conditions, a low ESR was observed at room temperature, even when whole blood was held in the ESR tube for up to 6 h (Figure 2a). Conversely, ESR significantly increased in the hemodilution groups, with rates three to four times faster than that of whole blood. However, the addition of extra Dextran‐40 greatly decreased ESR, which became more pronounced over time as the samples remained in the ESR tube. Notably, perfusate containing 12% Dextran‐40 showed the lowest ESR, suggesting that this concentration may be optimal (Figure 2b). For hemolysis assessment, we collected perfusate supernatants at various time points (0, 1, 2, 4, 6, and 8 h) using both the BBGs and the peristaltic pump system. Hemolysis became evident at 2 h in the peristaltic pump system and worsened over time (Figure 2c,d). In contrast, the BBGs system significantly alleviated hemolysis and consistently maintained low levels (Figure 2c,d). To evaluate the stability of the isolated organs in the perfusion system, we analyzed pH, lactic acid, glucose, and oxygen partial pressures (PaO_2_) in the perfusate through blood gas analysis conducted in rat livers ex vivo perfusion setup over a period of 6 h, with a flow rate of 5 mL/min. The BBGs system outperformed the peristaltic pump system by maintaining environmental homeostasis. It sustained pH levels within the physiological range of 7.2–7.45, whereas the peristaltic pump system showed pH levels dropping below 6.8 by 4 h (Figure 2e). Additionally, the BBGs system kept lactic acid concentrations below 5 mmol/L, in contrast to concentrations exceeding 5 mmol/L in the peristaltic pump system at 2 h (Figure 2f). Glucose concentration in the BBGs system showed only a slight increase, remaining below 18 mmol/L, while the peristaltic pump system recorded concentrations over 33 mmol/L by 6 h (Figure 2g). Furthermore, PaO_2_ levels remained relatively stable in the BBGs system, ranging from 150 to 180 mmHg, whereas the peristaltic pump system displayed wider fluctuations, with PaO_2_ ranging from 220 to 300 mmHg (Figure 2h).

### 2.3 BBGs promoted better cell adhesion and survival while maintaining cell morphology at a flow rate of 5 mL/min

**FIGURE 3:**
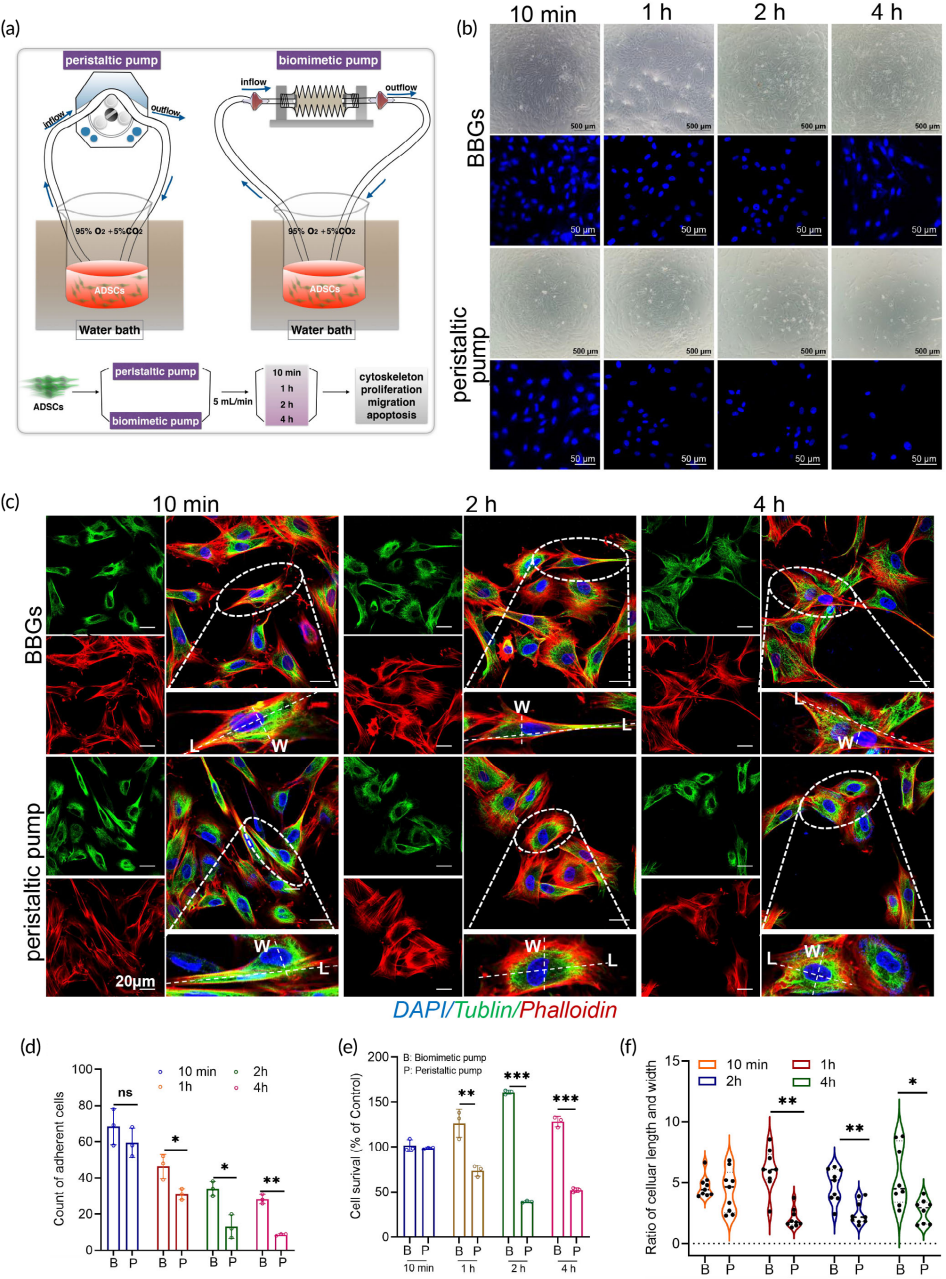
BBGs offered better adhesion and survival of cells with maintaining the cell morphology farthest at a flow of 5 mL/min. (a) Schematic of the methods used in this figure and Figures 4. (b) ADSCs observed by optical microscope (scale bars, 500 μM) and DAPI staining of the nucleus detected by confocal microscopy (scale bars, 50 μM) at 10 min, 1, 2, and 4 h by biomimetic pump or peristaltic pump at a flow of 5 mL/min, and (d) the quantitative analysis; *n* = 3. (c) Tublin/Phalloidin staining view (scale bars, 20 μM) and (f) the quantitative analysis of the ratio of cellular length and width of the perfused ADSCs; *n* = 8. (e) Cell survival of ADSCs, which has been perfused by biomimetic pump or peristaltic pump at 10 min, 1, 2, and 4 h and the subsequent culturing for 18–24 h; *n* = 3. L, length; W, width. Data represents the mean ± SD. **p < 0.01, ***p < 0.001. The experiments above were repeated three times.

For evaluating the cell survival and adhesion in extracorporeal perfusion systems, an initial in vitro experiment was developed. After circulating in the NMP systems (containing BBGs and peristaltic pump system) for 10 min, 1, 2, and 4 h, equal volumes perfusate initially containing identical quantities of ADSCs were collected (Figure 3a). These perfusates were subsequently cultured for 18–24 h for further experiments. In contrast to the peristaltic pump, BBGs demonstrated greater cell adhesion on the culture dish at 1, 2, and 4 h. Moreover, the protective effect of BBGs on cell adhesion became increasingly pronounced over time (Figure 3b–d). To further assess the impact of BBGs versus the peristaltic pump on cell proliferation, a CCK8 test was performed, revealing a higher cell survival rate with BBGs compared to the peristaltic pump (Figure 3e). Cell morphology undergoes corresponding changes during external stimuli, cell division, and differentiation, processes intricately linked to intracellular signaling regulation and dynamic cytoskeletal alterations. Consequently, the cytoskeleton, serving as the structural foundation of cell function, plays a key role in determining cellular functionality. As depicted in Figure 3c, after circulating in the perfusion system for 2 h or longer, compared to the BBGs group, ADSCs in the peristaltic pump group exhibited a rounder shape with a smaller cellular length‐to‐width diameter ratio (Figure 3f), indicating a shift in cellular function.

### 2.4 BBGs provided a more biocompatible microenvironment for cell proliferation with limited apoptotic cells at a flow rate of 5 mL/min

**FIGURE 4:**
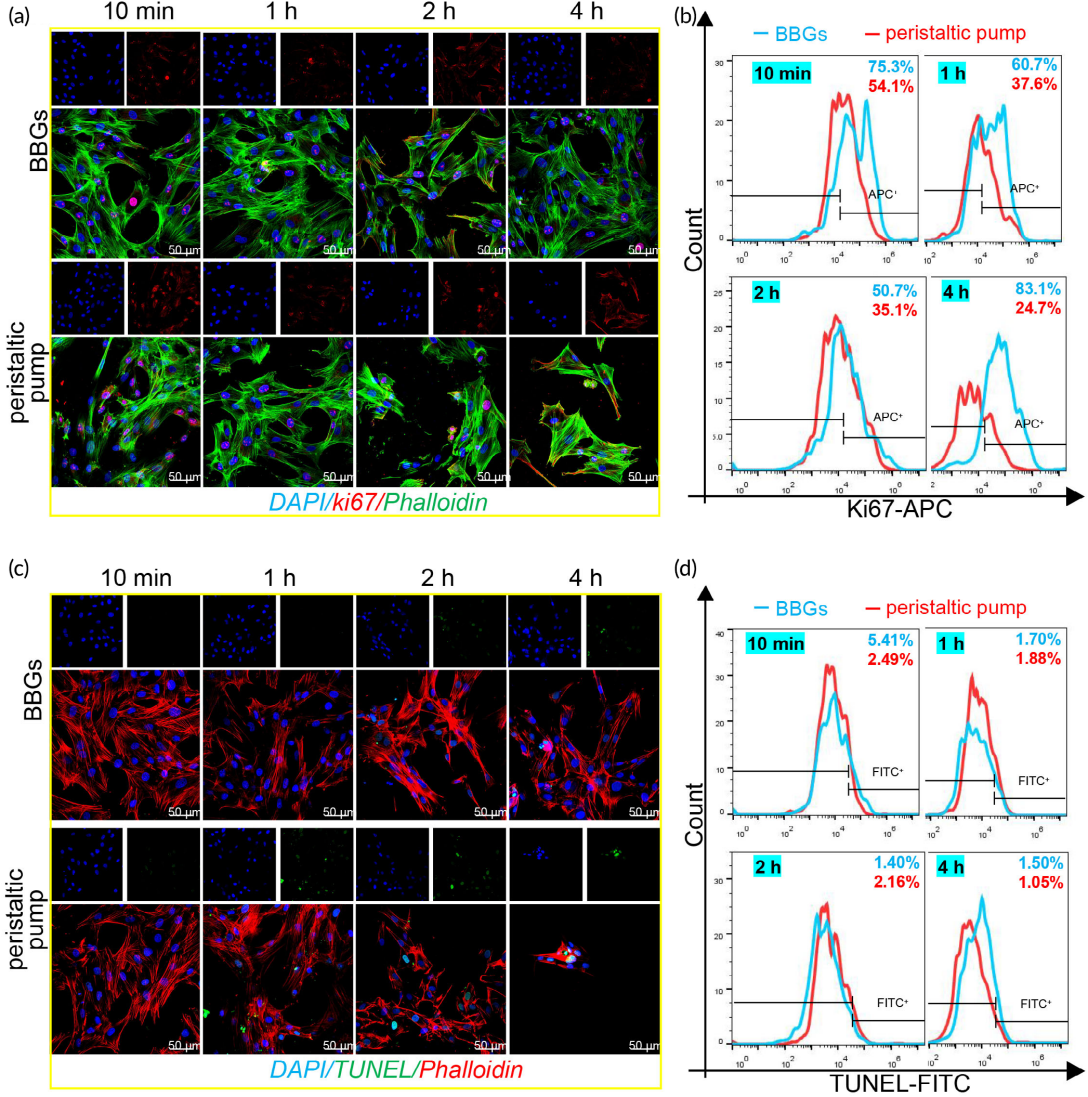
BBGs provided a more biocompatible microenvironment for cell proliferation with limited apoptotic cells at a flow of 5 mL/min. (a) ADSCs stained with Ki67 and Phalloidin for detecting cell proliferation observed by confocal microscopy at 10 min, 1, 2, and 4 h; (b) FCM analysis of Ki67 positive expression of ADSCs with biomimetic pump or peristaltic pump perfusing at 10 min, 1, 2, and 4 h; (c) ADSCs stained with TUNEL and Phalloidin for detecting cell apoptosis observed by confocal microscopy at 10 min, 1, 2, and 4 h; (d) FCM analysis of TUNEL positive expression of ADSCs with biomimetic pump or peristaltic pump perfusing at 10 min, 1, 2, and 4 h. Scale bars, 50 μM. The experiments above were repeated three times.

To further estimate cell proliferation and apoptosis, Ki67 and TUNEL staining were conducted on ADSCs that had been circulated in the perfusion system for 10 min, 1, 2, and 4 h. The results consistently demonstrated that BBGs maintained a higher cell proliferation rate throughout the entire perfusion period, with more cells adhering to the culture dish compared to those in the peristaltic pump group (Figure 4a). Similarly, flow cytometry (FCM) revealed a higher percentage of Ki67‐positive cells in the BBGs group, with proportions of 75.3%, 60.7%, 50.7%, and 83.1% at 10 min, 1, 2, and 4 h, respectively, compared to 54.1%, 37.6%, 35.1%, and 24.6% in the peristaltic pump group (Figure 4b). Apoptosis, a controlled process regulated by genes to maintain cellular homeostasis through programmed cell death, was investigated using TUNEL staining to detect nuclear DNA fragmentation during apoptosis. The results revealed no significant differences between the BBGs group and the peristaltic pump group, with both displaying only a small number of TUNEL‐positive cells in both groups (Figure 4c,d). This finding suggests that both perfusion systems have the potential to maintain intracellular homeostasis.

### 2.5 BBGs exhibited superior cellular adhesion and a higher cell survival rate at a flow rate of 50 mL/min

**FIGURE 5:**
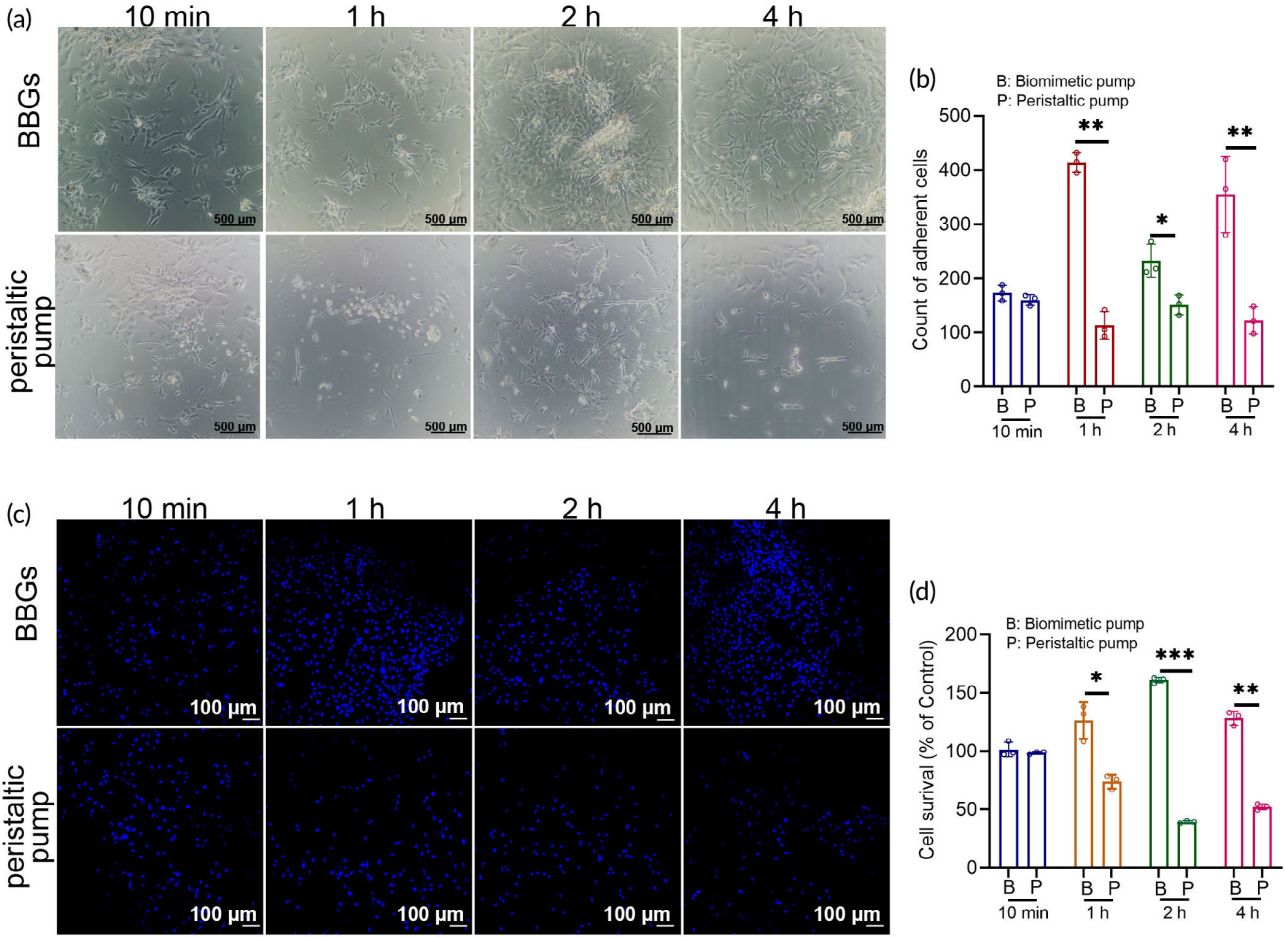
BBGs exhibited superior cellular adhesion and a higher cell survival rate at a flow rate of 50 mL/min. (a) ADSCs observed by optical microscope for detecting the number of cells at 10 min, 1, 2, and 4 h by biomimetic pump or peristaltic pump, Scale bars, 500 μM; (b) ADSCs stained with DAPI detected by confocal microscopy, and (c) the quantitative analysis for assessing cell adhesion at 10 min, 1, 2, and 4 h. Scale bars, 100 μM; (d) cell survival of ADSCs, which has been perfused by biomimetic pump or peristaltic pump at 10 min, 1, 2, and 4 h and the subsequent culturing for 18–24 h. Data represents the mean ± SD. *p < 0.05, **p < 0.01, ***p < 0.001. The experiments above were repeated three times.

To accommodate various applications requiring different flow velocities, the biological characteristics of ADSCs were evaluated under a flow rate of 50 mL/min, using procedures identical to those previously described in Section 2.1. Optical microscopy revealed results consistent with those obtained at a flow rate of 5 mL/min, showing only a small number of cells with a rounder shape adhered to the dish in the peristaltic pump group compared to the BBGs group (Figure 5a). Subsequently, DAPI staining was performed to visualize cell nuclei (Figure 5c) and quantitative analysis confirmed similar findings to those observed at a flow rate of 5 mL/min. After perfusing in the NMP at a flow rate of 50 mL/min, for more than 1 h, equal volumes of perfusion fluids were collected and analyzed. Compared to the peristaltic pump group, more adhesive cells were observed on the dish in the BBGs group (Figure 5b,c). To further evaluate cell survival in the different perfusion systems, a CCK8 test was conducted. The result revealed a superior promotion of cell proliferation in the BBGs group (Figure 5d).

### 2.6 BBGs exhibited superior cell morphology with a lower occurrence of apoptotic cells at a flow rate of 50 mL/min

**FIGURE 6:**
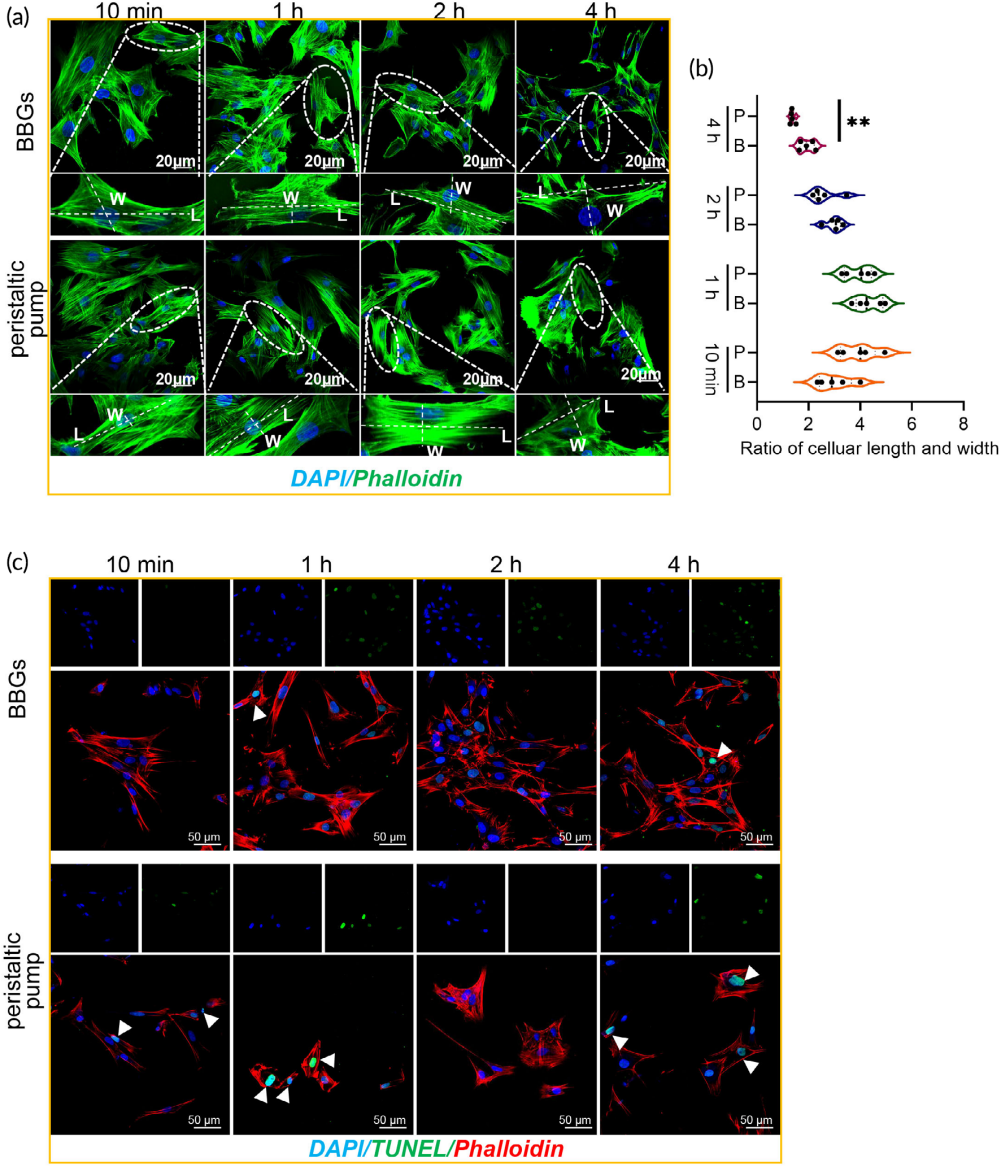
BBGs exhibited superior cell morphology with a small amount of apoptotic cells at a flow of 50 mL/min. (a) ADSCs stained with Phalloidin detected by confocal microscopy, Scale bars, 20 μM and (b) the quantitative analysis of the aspect ratio of cells. *n* = 5. (c) TUNEL and Phalloidin staining of the cells to estimate the apoptotic cells. White arrow: TUNEL positive cells. Scale bars, 50 μM. B, BBGs; L, length; P, peristaltic pump; W, width. Data represents the mean ± SD. **p < 0.01.

To assess cell morphology at a higher flow velocity, Phalloidin staining was conducted. The result revealed consistent finding with that obtained at a flow rate of 5 mL/min. After circulating in the perfusion system for more than 2 h, ADSCs in the peristaltic pump group tended to adopt a more spherical shape with a reduced cell length‐to‐width ratio compared to the BBGs group (Figure 6a,b), suggesting a change in cellular function. Despite the smaller number of cells adhering to the culture dish in the peristaltic pump group, both perfusion systems exhibited low cell apoptosis rates, which indicated the surviving cells maintained a favorable functional status (Figure 6c).

### 2.7 The supernatant collected from ADSCs perfused using BBGs showed reduced M1 macrophage presence and lower TNF‐α expression upon LPS stimulation

**FIGURE 7:**
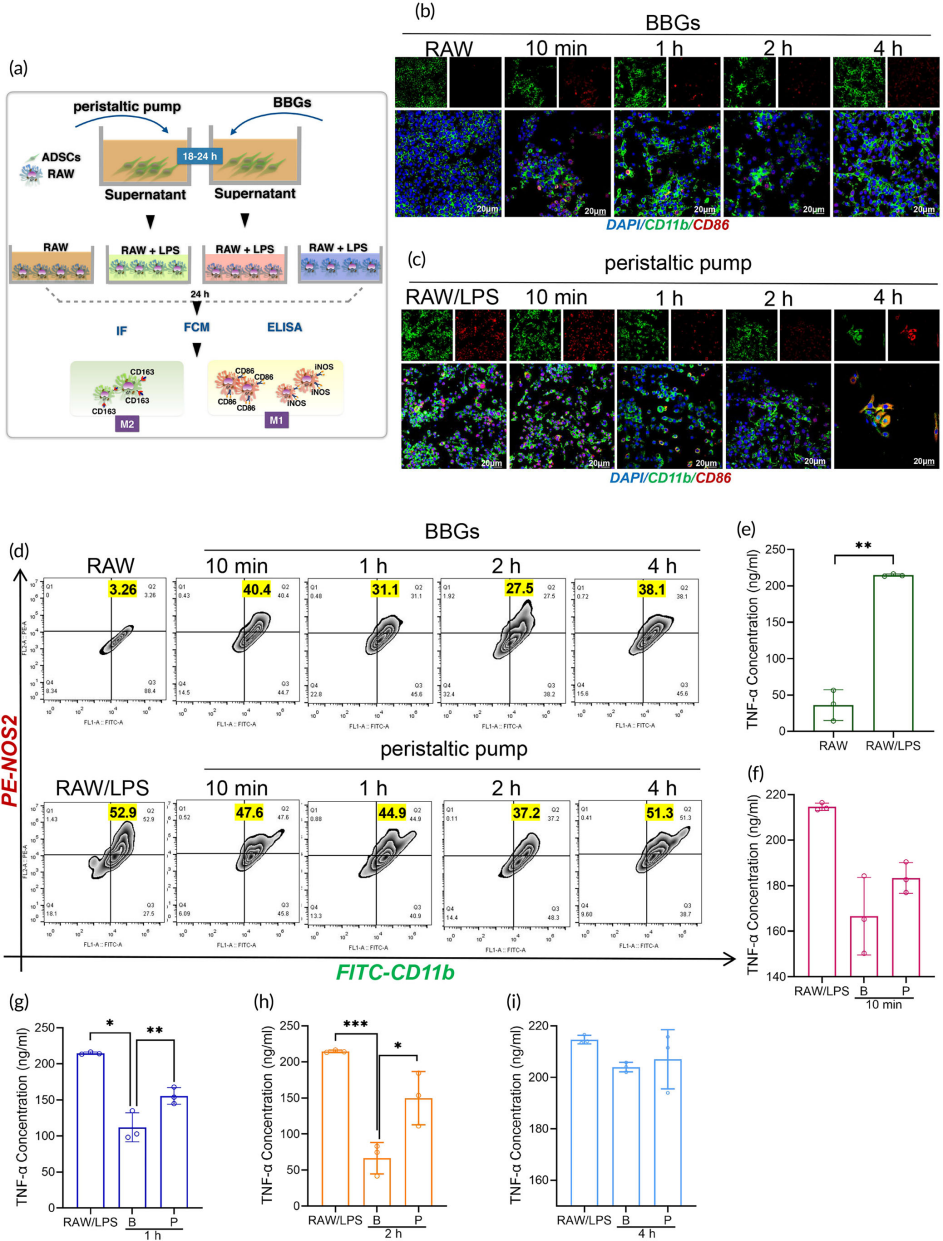
The supernatant from BBGs‐based perfused ADSCs exhibited reduced M1 macrophages under LPS stimulation, accompanied by lower TNF‐α expression levels. (a) Schematic of the methods used in this figure; CD11b and CD86 staining to detect M1 macrophage level with the treatment of the supernatant of (b) biomimetic pump or (c) peristaltic pump perfused ADSCs; (d) FCM analysis of CD11b^+^CD86^+^ cells at 10 min, 1, 2, and 4 h; (e)–(i) ELISA tests of the TNF‐α expression of supernatant of the ADSCs‐RAW 264.7 co‐culturing system. Scale bars, 20 μM. Data represents the mean ± SD. *p < 0.05, **p < 0.01, ***p < 0.001. The experiments above were repeated three times.

To explore the therapeutic effects of ADSCs for alleviating LPS‐induced inflammatory responses, ADSCs were collected after circulating in the perfusion system at different time points and cultured for 18–24 h (Figure 7a). Subsequently, supernatants containing secreted factors from ADSCs were collected and incubated with LPS‐stimulated RAW 264.7 for 24 h. In the presence of 1 μg/mL LPS, a significant portion of RAW 264.7 cells polarized towards the M1 phenotype, as evidenced by increased CD86 expression (Figure 7c). Upon treatment with ADSC supernatants, both experimental groups showed a reduction in M1 macrophages (Figure 7b,c). Specifically, the ratio of CD11b^+^NOS2^+^ cells decreased from 52.9% to 40.1%, 31.1%, 27.5%, and 38.1% in the BBGs group. In the peristaltic pump group, it decreased to 47.6%, 44.9%, 37.2%, and 51.3% (Figure 7d). In accordance with these findings, ELISA results for TNF‐α also indicated a significant reduction in its expression, particularly in the BBGs group. This reduction was most pronounced when using supernatants from ADSCs that had been circulated in the perfusion system for 1 and 2 h (Figure 7e–i).

### 2.8 Utilization of BBGs with extra ADSCs perfusion contributed to reduce the injury of livers from DCD

**FIGURE 8:**
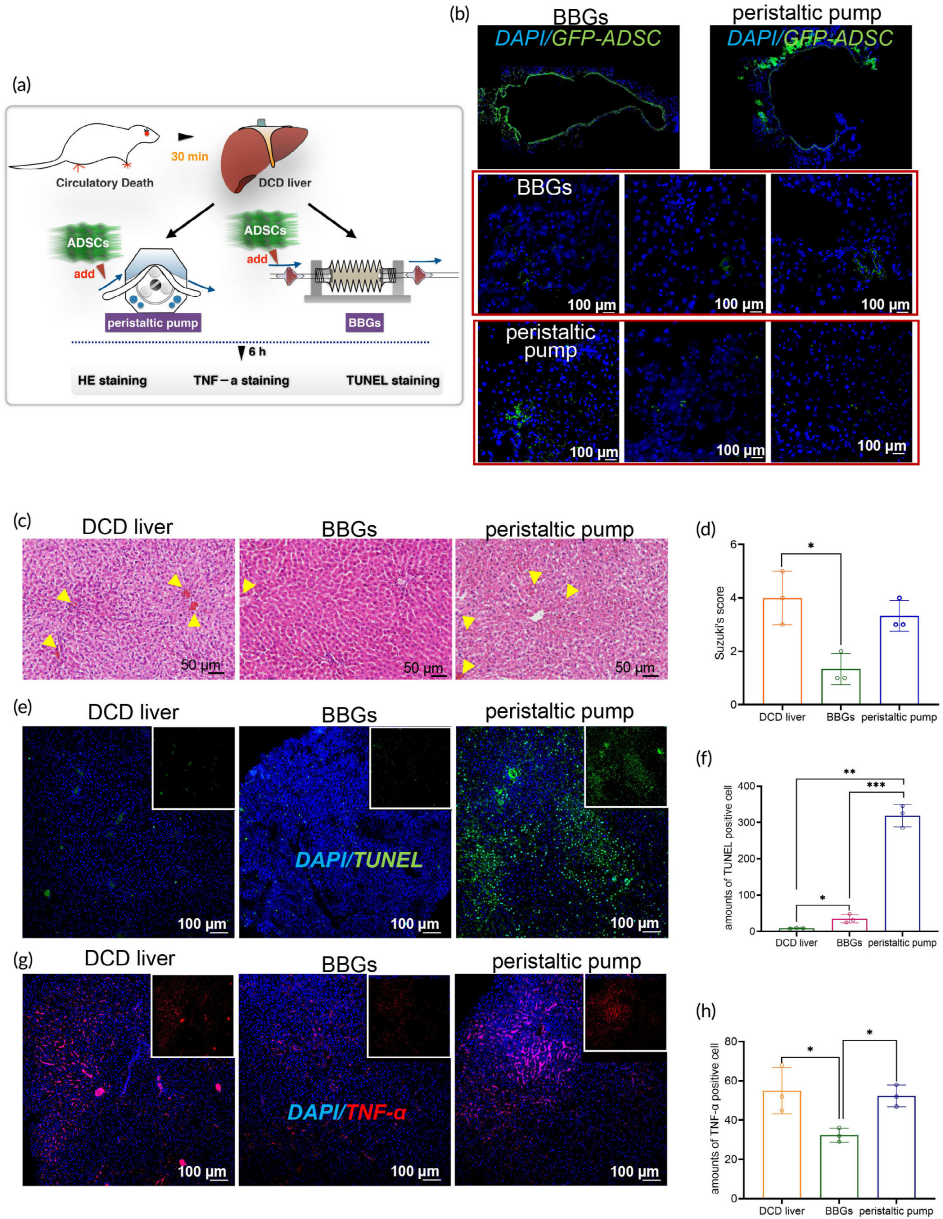
Utilization of BBGs with additional ADSCs perfusion helped reduce injury in DCD liver. (a) Schematic of the methods; (b) IF staining to detect the GFP‐ADCSs located around the central vein or hepatic lobule of the livers which have been perfused in the NMP system for 6 h, Scale bars, 100 μM; (c) HE staining, Scale bars, 50 μM and (d) Suzuki score of the pathological structure of the DCD liver. Yellow arrow: Hepatic congestion; (e) TUNEL staining and (f) the relative quantitative analysis to detect the apoptotic cells, Scale bars, 100 μM; (g) TNF‐α staining and (h) the relative quantitative analysis to detect the inflammatory conditions of the DCD livers. Data represents the mean ± SD. *p < 0.05, **p < 0.01, ***p < 0.001. The experiments above were repeated three times.

To further explore the influence of different NMP systems on the therapeutic efficacy of ADSCs for DCD livers, 2 × 10^7^ GFP‐labeled ADSCs were introduced into the perfusate and circulated for 6 h (Figure 8a). As shown in Figure 8b, GFP‐labeled ADCSs migrated from the vein to the hepatic lobules with no significant difference observed between the two experimental groups. However, the structure of hepatic lobules in the BBGs group (Figure 8c) notably differed from that in the peristaltic pump system, exhibiting reduced hepatic congestion, necrosis, vacuolar transformation, and edema, both interstitial and hepatocellular. Suzuki's histological grading also reflected these differences, with an average score of 1.3 in BBGs group compared to 3.3 in peristaltic pump group (Figure 8d), demonstrating a superior organ repair potential of BBGs. Owning to the IRI effect, more apoptotic cells were detected during the reperfusion of DCD livers in the NMP systems, whether using BBGs or peristaltic pump system. However, compared to the peristaltic pump group, BBGs group exhibited fewer TUNEL‐positive cells, suggesting the potential of BBGs in alleviating IRI injury (Figure 8e,f). Moreover, compared to the peristaltic pump, group the DCD liver perfused by the BBGs group showed significantly lower expression of the proinflammatory factor TNF‐α. (Figure 8g,h). In conclusion, extracorporeal liver perfusion with BBGs and additional ADSCs might represent an effective approach to reduce injury in DCD livers.

## 3 DISCUSSION

In this study, we introduced a novel designed BBGs to closely mimic physiological metabolism during extracorporeal perfusion of various isolated organs. The BBG system incorporates a flexible one‐way valve within the balloon pump, emulating the hydrodynamics of an in vivo heart valve. The balloon can elongate and compress within a range of 5 mm to 2 cm, enabling precise control over flow rates from 1 to 70 mL/min. This flexibility makes the BBG system suitable for accommodating the diverse perfusion requirements of various animal organs, especially when dealing with perfusate containing cells for extended culture or tissue repair. Additionally, the system features an intermittent oxygen supply controlled by a ventilator, which imparts lung‐like physiological functionality to the NMP system. This controlled breathing mechanism, involving both exhalation and inhalation, effectively mitigates the risk of hypoxia toxicity associated with continuous oxygen supply.^33^ The integration of a one‐way valve balloon pump and a ventilation control device within the BBG system represents a novel approach to maintaining microenvironment homeostasis. Although magnetic levitation pumps are widely adopted as the mainstream ventricular assist devices (VADs)^1^, ^3^, ^5^ due to their excellent blood compatibility,^12^ they still fail to meet the minimal flow velocity requirements for small animal studies. Our newly developed BBG system not only demonstrates superior blood compatibility but also offers a wide range of flow velocities, making it a promising solution for achieving improved organ perfusion in small animal models.

While stem cell therapy has found widespread application in treating various diseases, the mechanisms underlying these processes still require comprehensive analysis.^34^, ^35^ Some studies suggest that stem cells can be recruited to injured sites and differentiate into specific somatic cells. Others propose that exosomes or other secretory proteins released by stem cells play a critical role in regulating various physiological processes,^36^, ^37^ including inflammation inhibition, promotion of tissue regeneration, and maintaining homeostasis.^38^ Regardless of the mechanism, preserving the normal biological characteristics and cellular functions of stem cells is of paramount importance, particularly when dealing with IRI. In this study, compared to the peristaltic pumps, ADSC in the BBGs group showed less damage, including reduced cell disruption, enhanced cell proliferation, improved adhesion ability, and better maintained cell morphology. We speculate that the improved cellular behavior led to a quicker alleviation of ROS‐induced inflammatory reactions, which is particularly important in subsequent organ perfusion.

Extracorporeal organ perfusion provides a promising solution for extending organ preservation and offers an effective platform for the reparative regeneration of marginal organs. This approach holds potential for addressing the severe shortage of organ donors.^39^, ^40^, ^41^ Researches have demonstrated that Bone Marrow‐Derived Mesenchymal Stem Cells (BMMSCs) can alleviate the inflammatory response in DCD‐derived livers when subjected to the NMP systems.^27^ Livers transplantation after perfusion exhibit higher survival rates and fewer cell apoptosis events, demonstrating the effectiveness of this in vitro perfusion method in repairing marginal donor organs.

However, the unique liquid squeezing method employed by peristaltic pumps may negatively impact the maintenance of stem cell biological functions and have secondary effects on DCD liver transplantation. In this study, we found that the peristaltic pump system caused significant damage to stem cells, diminishing their capacity to alleviate macrophage‐induced inflammatory responses. The peristaltic pump group exhibited a significant increase in inflammatory damage and a higher number of apoptotic cells. In contrast, the reperfusion injury in the BBGs group was notably milder, highlighting its crucial in reducing initial IRI. These findings underscore the validity and significance of employing BBGs to alleviate early inflammatory responses in organ transplantation.

Previous studies have revealed that sustained exposure to high oxygen concentrations can lead to an overabundance of oxygen free radicals, thereby intensifying oxidative stress damage and causing organ injury.^18^, ^19^, ^20^ In our study, by simulating normal lung respiratory rates and regulating ventilation, we achieved a more stable perfusion system with consistent pH values, lactic acid concentrations, glucose levels and oxygen partial pressures. This stability was facilitated by shifting from continuous oxygenation to an inspiratory‐to‐expiratory ratio of 1:3. Compared to the peristaltic pump system, BBGs exhibited a superior ability to maintain internal environment homeostasis in the NMP system. The enhanced hemocompatibility of BBGs, crucial for preserving the integrity of cellular physiological functions, can be attributed to the inclusion of the flexible one‐way valve within the balloon pump and the integration of a ventilation control system.

## 4 CONCLUSION

The newly constructed BBGs, featuring a suction pump designed to mimic the cardiac cycle and an adjustable inspiratory‐to‐expiratory (IE) ratio, effectively mitigates acidosis resulting from continuous oxygen inhalation, largely protects the biological characteristics of the circulated ADSCs and improves their therapeutic effects on DCD liver. By modifying the pump's working mode from the powerful squeezing of peristaltic pumps to the flexible squeezing of balloon pumps, we have significantly reduced red blood cell damage. Additionally, we introduced an aeration regulation device into the BBGs perfusion system, improving the oxygen supply mode based on the bionic concept. However, evaluating the oxygen demand–supply balance at the level of isolated organs, tissues, and cells remains a significant challenge. This exploration is crucial for advancing in vitro organ perfusion studies and repairing marginal donor livers.

In addition, our perfusion system has some limitation. Unlike NMP systems used in large animal experiments or clinical trials, our system lacks an automated detection module. Currently, we manually collect perfusion fluid at fixed intervals and rely on additional equipment for biochemical and blood gas analyses, which introduces a delay in organ status and feedback. Our future efforts will focus on integrating an online automatic biochemical detection module into the perfusion system.

The development and application of the BBG system primarily aim to advance basic scientific research on in vitro small animal organs. In our upcoming work, we plan to design a perfusion platform tailored for large animal organs. Insights gained from the BBG system's power pump and respiratory functionalities will inform the construction of these larger‐scale perfusion systems. This progression holds promise for facilitating the repair of marginal donor organs and may eventually pave the way for clinical translation.

## 5 MATERIALS AND METHODS

### 5.1 Materials

Dextran‐40 and Triton X‐100 were purchased from Macklin (CAS: 9004‐54‐0, D807616). Ki67 Polyclonal Antibody, Anti‐mouse Alexa Fluor 488, Anti‐mouse Alexa Fluor 555, Anti‐rabbit Alexa Fluor 488, and Anti‐rabbit Alexa Fluor 555 were purchased from Invitrogen. Cell Counting Kit‐8 (CCK8) and Lipopolysaccharides (LPS) were purchased from Solarbio. FITC‐Rhodamine, rabbit Anti CD86 Antibody, and TNFα Elisa kit were purchased from abclonal. FITC‐TUNNEL was purchased from Meilunbio. Rabbit CD11b Antibody was purchased from Biolengend. Mouse TNF‐α Antibody was purchased from Santa Cruz.

### 5.2 BBGs fabrication

BBGs was constructed by a set of one‐way valve sacculus pump and a ventilator. The sacculus, individually designed for different flow velocities for various in vitro organs perfusion, was constructed by precise 3D‐printing technology with Thermoplastic Polyurethanes (TPU). TPU was chosen for its mechanical flexibility, transparency, and elasticity.

### 5.3 Animals

For liver transplantation‐related experiments, healthy, male Sprague–Dawley (SD) rats, aged 8–10 weeks and weighing between 220 and 250 g, were selected due to their stable hormonal profiles and ease of handling. These animals were sourced from Beijing HFK Bioscience Co., Ltd. (Beijing, China, Certificate Number: SCXK Jing 2019‐0008). All animal experiments were approved by the Institutional Animal Care and Use Committee of Nankai University (Permit Number: 2021‐SYDWLL‐000393). Animals were received humane care in compliance with the Principles of Laboratory Animal Care formulated by the National Society for Medical Research, with a relative humidity between 40% and 50%, a temperature of 26°C, freely access to water and food and a 12‐h light/dark cycle.

### 5.4 Extracorporeal perfusion phase

SD male rats were anesthetized with 4% Chloral hydrate (100 g/0.2 mL) and Zoletil 50 (100 g/0.02 mL); then, systemic heparinization was performed by injecting 500 units of heparin through the tail vein. 5 mL of blood were taken from each rat's inferior vena cava for subsequent sedimentation and hemolysis testing, and as an important component of the perfusate. Next, the abdominal cavity was opened and the hepatic artery (HA), the portal vein (PV), and the bile duct were separated. The hepatic artery was ligatured, and both the hepatic artery and bile duct were intubated with 3 mm × 3.8 mm and 0.28 mm × 0.64 mm tubes, respectively. The livers were flushed with cold heparin‐saline through the HA cannulas until the lavage solution ran clear. The perfusion system was primed with 40 mL perfusate with 40% oxygen, then the livers were connected to the NMP through the HA cannulas at a flow rate of 4 mL/min for 6 h. For BBGs, I/R ratio of the ventilator was adjusted to 1:3. Perfusate samples were taken at 0, 1, 2, 4, and 6 h. Blood gas monitor (i‐STAT300G, G4^+^, USA) was applied to evaluate the value of pH, glucose, lactate and partial pressure of oxygen (PaO_2_) at room temperature.

### 5.5 Hemolysis and erythrocyte sedimentation rate assessment

Mechanical crushing could lead to erythrocyte lethal destruction and the subsequent release of a large amount of hemoglobin into the perfused solution, causing the perfused supernatant color turn to red. The whole blood was added to the perfusion solution to maintain a hematocrit of 10%; then, 50 mL of the perfusion solution was taken and transferred into two perfusion systems at a working flow rate of 5 mL/min. One milliliter perfusate was collected at the time point of 0, 1, 2, 4, 6, and 8 h. The collected samples were centrifuged at 2500 rpm for 5 min to separate the supernatant. The absorbance value was measured at an OD value of 576 nm. The hemolysis rate (%) was calculated using the following formula: Hemolysis Rate (%) = [(OD value of sample − OD value of negative)/(OD value of positive − OD value of negative)] × 100%. The 0 h group served as negative control, and the group with 0.1% Triton‐100 water added was used as the positive control. Dextran‐40 was added into the perfusate to increase viscosity and colloid osmotic pressure maintenance for decreasing ESR. For ESR assessment, 8%, 10%, and 12% Dextran‐40 were added into the perfusate and then transferred into ESR tubes; the ESR tubes were placed vertically on the ESR shelf and the distance at which RBCs sank at 1, 2, and 6 h were recorded.

### 5.6 ADSC biology testing

#### 5.6.1 Cell culture

ADSCs were harvested from 4‐week‐old male SD rats or GFP‐transgenic rats as has been reported.^42^ Briefly, bilateral inguinal fat pads were taken from the disinfectant rats and then washed in phosphate‐buffered saline (PBS) containing 100 U/mL penicillin and 100 μg/mL streptomycin for 10 min. The harvested tissues were cut into scraps and applied to enzymatic treatment using 0.075% collagenase type I at 37°C for 40 min. Following the equal volume PBS was added, the tissues were centrifuged at 1000 rpm for 5 min and the pellet at the bottom of the centrifuge tube was retained for subsequent cell culture.

#### 5.6.2 Cell perfusion

A total of 4 × 10^7^ ADSCs were introduced into the NMP system in a final volume of 50 mL, with 90% oxygen was supplied. NMP was operated at two different flow rates: a low flow rate of 5 mL/min and a high flow rate of 50 mL/min. Perfusate samples containing cells were collected at four time points: 10 min, 1, 2, and 4 h.

#### 5.6.3 Cell proliferation detection

Perfusate samples (0.5, 1, 2, and 4 mL) obtained at the specified time intervals (10 min, 1, 2, and 4 h) were individually centrifuged at 2500 rpm/min for 5 min. After centrifugation, the cell pellets were resuspended in 100 μL of culture medium and subsequently seeded onto 96‐well plates. Following an incubation period of 18–24 h, a Cell Counting Kit‐8 (CCK8) assay was performed to assess cell viability. Cell survival was calculated using the following formula: (OD value of the sample − OD value of the blank)/(OD value of the control − OD value of the blank) × 100%.

#### 5.6.4 Immunofluorescence and flow cytometry measurement

One, 2, 4, and 8 mL perfusate were taken and centrifuged at 2500 rpm/min for 5 min at 10 min, 1, 2, and 4 h, separately; then, the cell sedimentations suspended in 200 μL medium were seeded on 48‐well plates. After incubated for 18–24 h, cells were washed with PBS for three times and then sequentially incubated with 4% paraformaldehyde (PFA) for 10 min at room temperature. 0.1% Triton for 15 min at room temperature and 5% BSA for 30 min at 37°C. Primary antibodies were added into the samples and incubated overnight at 4°C; with the PBS washed for three times, secondary antibodies were added into the samples and incubated at room temperature for 1 h. At last, DAPI was added to stain cell nuclei. For FCM evaluation, PE‐NOS2 was applied to detect the M1 polarization macrophages.

#### 5.6.5 Macrophage polarization estimation

Mouse monocyte macrophage RAW264.7 cells were seeded on 48‐well plates (10^5^ cells/well). Supernatants of the perfused ADSCs, which had been incubated for 18–24 h, and 1 μg/mL lipopolysaccharides were supplemented into the macrophage culture medium to detect macrophage polarization. After 24 h, supernatants were collected for later TNFα test and the cells were collected for further IF and FCM assessment.

### 5.7 Livers from DCD model construction and perfusion

Prior to the experiment, 200 units of heparin sodium were injected into the tail vein of the SD rats. SD rats were sacrificed to result in circulation death and 30 min later, livers were harvested as DCD livers. The surgical procedure and preparation of the perfusion system are consistent with those described in Section 5.4. A total of 2 × 10^7^ ADSCs were added into the liver via PV. The perfusion was carried out at a flow rate of 4 mL/min for a duration of 6 h.

### 5.8 Histology and immunofluorescence

Following perfusion, the DCD livers were fixed in 4% paraformaldehyde (PFA) for a period of 24 h. After dehydrated and embedded in paraffin, livers were cut into 5 μm thick paraffin sections. The sections were subjected to antigen retrieval immediately after deparaffinization, followed by permeabilization with 1% Triton X‐100, and blocking was performed with 5% bovine serum albumin. Slices were then incubated with TUNNEL or TNFα at 37°C for 2 h. Followed by washing, the slices were incubated with each of the corresponding secondary antibody and DAPI. The immunofluorescence expressions were detected by Confocal laser scanning microscopy (FV100, Olympus, JP).

### 5.9 Statistical analysis

All experiments were performed in three replicates. The significance was analyzed using a one‐way analysis of variance (ANOVA) with Tukey's post hoc test. The data were presented as mean ± standard deviation (SD). Statistical significance was set at *p* < 0.05.

## AUTHOR CONTRIBUTIONS

**Tingting Lan:** Conceptualization; data curation; formal analysis; funding acquisition; investigation; project administration; writing – original draft. **Mingxing Yu:** Conceptualization; data curation; formal analysis.

## CONFLICT OF INTEREST STATEMENT

The authors declare no competing interests.

### PEER REVIEW

The peer review history for this article is available at https://www.webofscience.com/api/gateway/wos/peer-review/10.1002/btm2.10724.

## Supporting information


**FIGURE S1:** BBGs fabrication. (a) Designed BBGs module; (b) general view of the NMP.

## Data Availability

The original contributions presented in the study are included in the article. Further inquiries can be directed to the corresponding author.
