# Supplementary figures and images for: A novel cytoprotective organ perfusion platform for reconstructing homeostasis of DCD liver while alleviating IRI injury
Source: Bioeng Transl Med. 2024 Sep 23;10(1):e10724. doi: 10.1002/btm2.10724 (PMC11711209; doi:10.1002/btm2.10724)

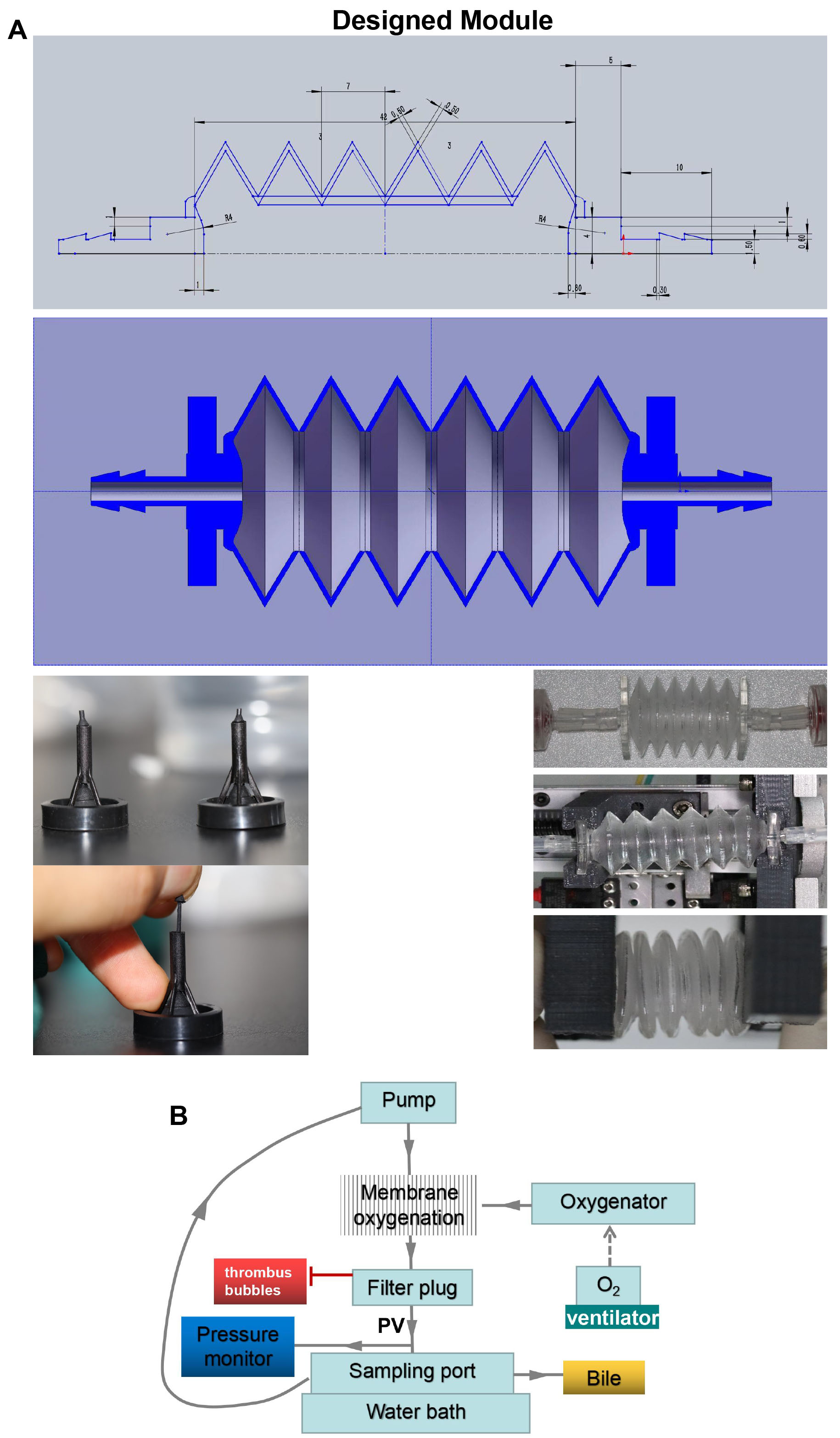

Supplement: Supplementary file 1 — FIGURE S1: BBGs fabrication. (a) Designed BBGs module; (b) general view of the NMP. [file BTM2-10-e10724-s001.jpg]
